# Identification of MVOCs Produced by *Coniophora puteana* and *Poria placenta* Growing on WPC Boards by Using Subtraction Mass Spectra

**DOI:** 10.3390/ijerph16142499

**Published:** 2019-07-13

**Authors:** Mateusz Kozicki, Anna Wiejak, Michał Piasecki, Alicja Abram

**Affiliations:** 1 Department of Thermal Physics, Acoustic and Environment, Building Research Institute, 00-611 Warsaw, Poland; 2 Department of Construction Materials Engineering, Building Research Institute, 00-611 Warsaw, Poland

**Keywords:** MVOC, wood-polymer composite, *Basidiomycetes*, TD-GC/MS

## Abstract

Volatile fungal metabolites are responsible for various odors and may contribute to a “sick building syndrome” (SBS) with a negative effect on the heath of building. The authors have attempted to fill the research gaps by analyzing microbial volatile organic compounds (MVOCs) originating from representatives of the *Basidiomycetes* class that grow on wood-polymer composite (WPC) boards. WPCs have been analyzed as a material exposed to biodeterioration. Indoor air quality (IAQ) is affected by the increased use of WPCs inside buildings, and is becoming a highly relevant research issue. The emission profiles of MVOCs at various stages of WPC decay have been demonstrated in detail for *Coniophora puteana* and *Poria placenta*, and used to set the European industrial standards for wood-decay fungi. Differences in the production of MVOCs among these species of fungi have been detected using the thermal desorption-gas chromatography/mass spectrometry (TD-GC/MS) method. This study identifies the production of alcohols, aldehydes, ketones, carboxylic acids and other compounds during one month of fungal growth. The identified level of metabolites indicates a relation between the level of air pollution and condition of the WPC material, which may become part of IAQ quantification in the future. The study points to the species-specific compounds for representatives of brown and white-rot fungi and the compounds responsible for their odor. In this study, 1-Octen-3-ol was indicated as a marker for their active growth, which is also associated with SBS. The proposed experimental set-up and data analysis are a simple and convenient way to obtain emission profiles of MVOCs from microbes growing on different materials.

## 1. Introduction

Biodegradation of wood and polymeric materials involves a series of biological and chemical processes, resulting in the destruction of polymer chains and wood structures when exposed to fungal hyphae. During this process, different types of fungi produce a unique grouping of microbial volatile organic compounds (MVOCs), which can also be used for species identification. This phenomenon indicates the possibility of using MVOCs as detection markers for wood decay fungus [1].

There are two common methods for the collection of MVOCs. The first entails a collection of a whole air sample in a stainless-steel sampling vessel, such as a canister or mini-can, using passive diffusion devices. The second method involves passing whole air through an air sampler on a solid sorbent inside a glass tube. The absorbent tube method is markedly more sensitive than the solvent extraction technique, while analysis of desorbed compounds using TD-GC/MS is the most commonly used method for detecting, identifying, and quantifying MVOCs. Moreover, this method is very sensitive in complex environmental matrices where MVOC levels are low [2,3].

The *Basidiomycetes* class is one of the most potent degraders of cellulose. These indoor, wood-destroying fungi are commonly found in European buildings [4]. The development of fungi causes changes in the chemical, physical and mechanical properties of wood or wood-based materials, which has a negative impact on their technical properties, especially durability. Resistance of wood-polymer composite (WPC) materials to an attack by *Basidiomycetes* determines the ENV 12038:2002 standard [5]. Degradation of the cell-wall structure in wood occurs under the influence of enzymes secreted by the hyphae of mycelium: cellulase and ligninase. The metabolism and MVOCs produced by wood-rotting *Basidiomycetes* [6,7,8] have been investigated extensively. However, there is a lack of research focused on profiling the MVOCs produced by *Basidiomycetes* colonization of WPC.

Composite materials are a combination of two or more basic constituent materials, which have substantially different chemical or physical properties [9]. In WPC, polymers form a continuously binding matrix that surrounds the wood components. Matrix polymers are materials with a high molecular mass, such as polyethylene (PE), polypropylene (PP), polyvinyl chloride (PVC), polystyrene (PS) and polylactide (PLA), which possess desirable physical properties, such as viscoelasticity or thermoplastic properties. Wood fillers perform a reinforcing role thanks to their strength and hardness [10,11]. Kim and Pal [12] indicate two main reasons for adding wood to polymers: to lower the price of the final product and to reduce dependency on mineral oil-based products. In addition, modification of wood fibers significantly reduces water absorption capacity, which is important for resistance to biological agents. The most widespread indoor applications of WPCs are railings, molding, trim, window and door frames, sheathings, elements of roof and floor construction, and indoor furniture. The most common outdoor uses are deck floors, landscaping timbers, cladding and siding, fences and park benches [13,14,15,16].

Several hundred volatile organic compounds have been detected in residential indoor air. When a residential area is infested with microorganisms, their metabolic processes produce microbial volatile organic compounds (MVOCs). These microbial metabolites are released during growth of fungi, mold and other microbes. The diversity of MVOCs is large, estimated at over a thousand different compounds, which are a complex mixture of organic compounds, including fatty acids and their derivatives, such as hydrocarbons, aliphatic alcohols, aldehydes and ketones, aromatic compounds or terpenes and terpene derivatives [17]. Garcia et al. [2] summarized the most common MVOCs reported for indoor and outdoor environments. The data presented in this work show that MVOC concentrations are higher in indoor environments because they remain closed and ventilation rates are lower compared to outdoors. In turn, Korpi et al. [18] have found that single MVOC levels in indoor environments range from a few ng/m^3^ up to 1 mg/m^3^.

MVOCs are products of the primary and secondary metabolism of organisms, such as fungi and bacteria. In primary metabolism, organisms create MVOCs as by-products, breaking down food in the environment to synthesize DNA, and amino and fatty acids used to maintain cell structures. The number of primary metabolites is relatively small, and their biosynthesis is well known. Secondary metabolism covers chemical compounds that may be necessary in order to survive in the environment and cope with various hazards. Secondary metabolites perform adaptive functions, playing a key role in communication between fungi and the environment and they are unique to individual species. They do not perform basic biological functions and many of them have a complex and branched biosynthetic pathway [18]. In other words, secondary metabolism consists of subsequent downstream reactions, while the production of secondary metabolites is often species-specific or restricted to a phylogenetic group [19,20].

A World Health Organization (WHO) report on biological agents [21] and other sources [22,23,24,25,26] describes the results of research designed to identify which MVOC arise from the fungal and bacterial growth on the surfaces of various building materials and microbiological media.

The growth of fungi on building materials generates emissions of both reactive and nonreactive volatile organic compounds. These emissions may contribute to “sick building syndrome” (SBS) [27,28]. SBS is a situation in which building occupants experience acute health and comfort effects, such as irritation of the eyes, nose and throat. It has been shown that some compounds associated with SBS are related to those produced in the presence of *Basiciomecytes* growing on WPC. In this study, MVOCs have been measured in order to identify microbial growth in building construction in the case of WPC, as described in the following sections.

## 2. Materials and Methods

Two separate composite boards per fungi species were placed on a sterile malt-extract agar medium in each Kolle flask (experimental chamber), and two sterilized samples in other Kolle flasks served as controls (control chambers). The experiment was carried out simultaneously using two experimental chambers for each fungal species. Only the MVOCs emitted in both experimental emission chambers for the same mold strain are reported in this study.

### 2.1. Experimental Design

**Figure 1:**
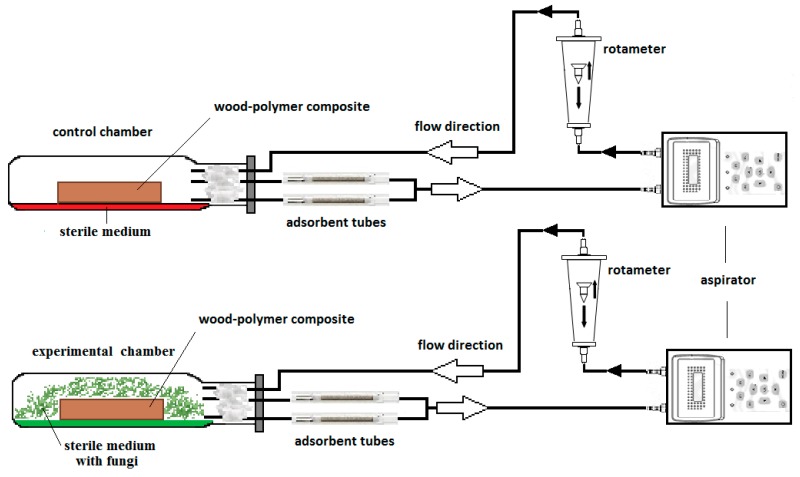
Schematic diagram of enclosed experimental set-up for collecting microbial volatile organic compounds (MVOCs) produced by *Basidiomycetes* grown on wood-polymer composite (WPC).

To obtain emission profiles for the MVOCs, an experimental set-up was constructed (Figure 1). Analyses were performed using the aspiration air sampling method. MVOCs were collected on adsorbent glass tubes filled with Tenax TA resin by air suction using an aspirator. The sample loop contained a rotameter to measure airflow. This was then transferred back into the growth chambers, thus forming a closed sample system.

### 2.2. Choice of Fungi Species

Fungi from the *Basidiomycetes* class are the most dangerous factor in the destruction of wooden elements in objects. The emission profiles of MVOCs at various stages of WPC decay have been demonstrated in this study for *Coniophora puteana* and *Poria placenta*, which the European industrial standards for wood-decay fungi are based upon. Moreover, there is lack of literature relating to MVOC profiles produced from *Basidiomycetes* colonization of WPC.

### 2.3. Fungal Strains, Growth Conditions and Culture

The wood-decay fungi strains used in this study were brown-rot fungus (Coniophora puteana (Schumacher ex Fries) Karsten (BAM Ebw. 15)) and white-rot fungus (Poria placenta (Fries) Cooke sensu J. Eriksson (FPRL 280)). They were separately cultured in Kolle flasks on malt extract agar (MEA) which consisting of 25 g/L agar and 40 g/L malt extract (Zakład Enzymów i Peptonów BTL). Each bottle had a volume of 250 mL.

**Figure 2:**
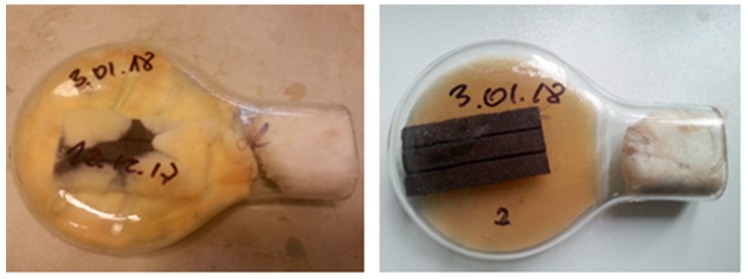
Kolle flask with *Coniophora puteana*—experimental chamber (on the left) and control chamber (on the right) after two weeks of incubation; photo taken by one of the contributing authors.

Pilot studies were performed on different WPC types; however, it was difficult to detect differences between these, because emissions were conditioned mainly by fungal metabolites. Finally, WPCs with high-density polypropylene (HDPP) matrices were chosen as the cultivation substrate. The composite materials consisted of 51% wood fibers and 49% HDPP. WPCs were cut into 80 mm × 40 mm × 6 mm cubes and put into Kolle flasks (Figure 2). MVOC emissions from the uncontaminated fungal composites on the agar medium blanks (in the control chambers) were used as background mass spectra.

The Kolle flasks were incubated for four weeks in a dark incubator at a temperature of 22 ± 2 °C and humidity of 70 ± 5%. The Kolle flasks were sealed tightly with cotton wool to enable exchange of inside and outside air. During incubation, MVOCs were taken from the flasks (adsorption tubes were inserted next to the cotton wool) after one day, two weeks and one month. Experimental conditions were maintained at a constant level throughout the whole incubation period.

### 2.4. Sampling and Analysis

Thermal desorption tubes are the most common sampling technique used for environmental MVOCs; these tubes allow detection of directly desorbed compounds into the GC/MS and avoids sample preparation. Air samples from the incubation chambers were adsorbed in Tenax TA, which is preferred because of its efficiency in trapping low concentrations of organics from air samples and because it is chemically inert, thermally stable and has good storage stability [3,29]. The Tenax desorption tube technique requires a short sampling time and low sample volume, thereby obtaining high sensitivity [2]. Moreover, Tenax TA has a low affinity for water, which makes it ideal for use in humid environments [7]. The air was collected over a period of 8 min at an air flow rate of 2 L/h (volume of the Kolle flask).

### 2.5. Thermal Desorption-Gas Chromatography/Mass Spectrometry (TD-GC/MS)

The separation process and these analyses of volatile compounds were achieved using a gas chromatograph equipped with a gas chromatography mass spectrometer (model GCMS-QP2010, Shimadzu, Tokyo Japan). The following GC oven temperature methodology was applied: an initial temperature of 40 °C for 5 min, then 10 °C increases per minute up to 260 °C, with a final temperature of 260 °C for 1 min. The transfer line temperature was held at 250 °C, whereas the source temperature was kept at 220 °C. Chromatographic separation was performed on a ZB-5MSi capillary column (30 m, 0.25 mm ID, 1 µm df), which favors identification of nonpolar compounds. The 1:10 split- ratio injection mode was applied.

Volatile organic compounds were thermally desorbed using a thermal desorption (TD 20, Shimadzu) cold-trap injector (cryo-focused at −18 °C). The tubes were heated at a temperature of 280 °C. Adsorbed compounds were then released into the flow of helium carrier gas to the GC/MS, which was used for MVOC identification and quantification.

The GC/MS analysis system includes an odor database (Smart Database; Shimadzu Corporation) with parameters and sensory information (such as types of odors and odor threshold values) for the primary compounds that cause odors.

### 2.6. Calculation Method for MVOC Concentrations

GC/MS software has the ability to subtract spectra. If the background spectra (from the control chamber) are subtracted from the spectra under investigation (experimental chamber), clearer spectra can be obtained. This can be done automatically or manually. For the data presented in this study, the automatic method was applied. Ions present in the background spectrum have been reviewed for possible background contamination or coeluting peaks. Taking into account the assumptions of the experiment, there are some circumstances in which this gives the best approximation.

### 2.7. Reference Compounds

Volatile compounds were identified by comparing the retention times of chromatographic peaks with those of reference compounds and by searching the NIST 2014 mass spectral database. All volatile compounds with mass spectra match factors *p* ≥ 90% were considered identified. The reference standards were analyzed in the same conditions as the experimental tubes. The reference compounds used to confirm the identity of the metabolites were as follows: alcohols (propanol, 2-hexanol and 2-heptanol); aldehydes (propionaldehyde, hexanal and 1-heptanal); carboxylic acid (propionic acid, hexanoic acid and heptanoic acid); ketones (acetone, 2-hexanone, 2-heptanone) and toluene. A solution of 200 µg/mL of each compound was produced by gravimetric measurement and dissolving the individual substances in methanol (CPAchem Ltd., Stara Zagora, Bulgaria). Standard solutions were prepared by injecting the compounds into the inert gas flow in the tube; the spiked adsorbent tubes were then thermally desorbed in the same conditions as the samples [30,31].

Compound-specific response factors were used to estimate the MVOC concentrations, determined by a certificated standard solution of 200 µg/mL foreach compound (CPAchem Ltd.). Identified compounds were quantified using their individual response factors when the reference compound was available. Concentrations of MVOCs other than the reference compounds were estimated by referring to compounds belonging to the same homologous series.

## 3. Results

### 3.1. Identification of MVOCs

**Figure 3:**
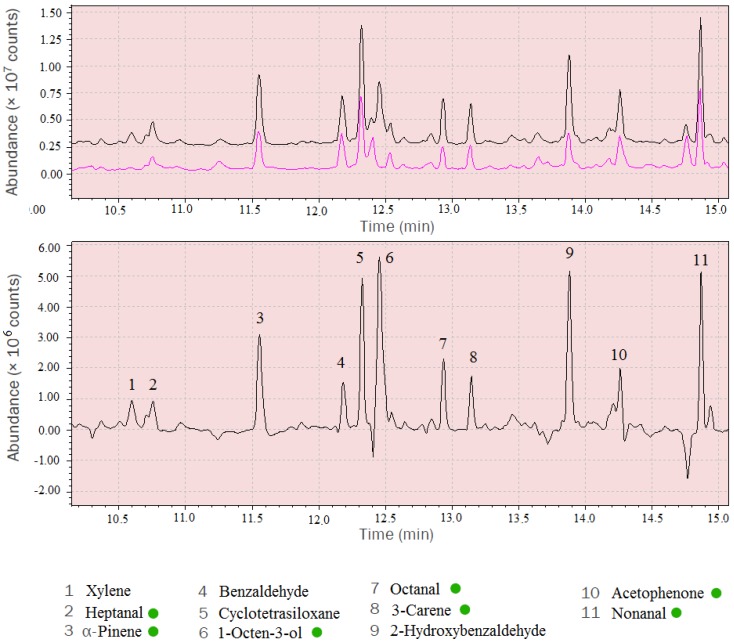
Comparison of control chamber spectrum (red) with experimental chamber (black) (**A**) and the subtracted mass spectrum with identified compounds (**B**). MVOCs are marked by green dots.

Mass spectra of the MVOCs were obtained using spectrum subtraction. The analysed mass spectra were obtained by subtracting background mass spectra (control chamber) from the spectra under investigation (experimental chamber) (Figure 3).

Table 1 contains the indicative concentration of MVOCs released from the culture of Coniophora puteana and Poria placenta growing on the WPC and agar-based medium. MVOCs are divided due to their chemical nature (alcohols, aldehydes, carboxylic acids, terpenes, ketones and sulphur compound). The concentrations measured in this study are equilibrium values obtained at samplingpoints, attributed by the exchange of outside and inside air in the Kolle flasks.

### 3.2. Identified Odor Compounds

In accordance with the WHO report [21], there is a strong probability that the intensity of the odor emitted by the MVOCs (e.g., an earthy, musty, fruity or mushroom-like smell) is responsible for the general smell of the building. Many worldwide committees are working on unifying and adopting the level of odor intensity emitted by indoor MVOCs as an indicator of air quality perceptible by humans [21].

The musty-type mold odor of experimental cultures cannot be thus based on the presence of a single substance but rather on the coexistence of several relevant compounds. Many MVOCs have distinctive odors, perceptible by the human sense of smell. Excessive humidity supports their growth and, consequently, smell. Odors derived from the compounds identified in this study are tabularized in Table 2.

## 4. Discussion

### 4.1. Analysis of MVOC Emission Profiles

The emissions of MVOCs change in accordance with the fungi type and their growth phase. Emissions also depend on existent environmental conditions, such as moisture, temperature and substrate composition [26]. In a nutrient-rich environment, the microbe produces a particular MVOC profile, which corresponds to stress-free growth, but in stressed environmental conditions (with competition or limited nutrients), a different MVOC profile may be produced, which enables adaptation to changing resource availability [19].

Fungi produce a number of different MVOCs including alcohols, aldehydes, carboxylic acids, terpenes, ketones and sulphur compounds. These compounds have been reported as fungal second metabolites in numerous former studies [32,33,34].

In each case, after one day of incubation, 1-octen-3-ol was detected in the range from 50 to 100 µg/m^3^. This unsaturated secondary alcohol gives a distinctive earthy mushroom odor (see Table 2) and is one of the most common compounds detected in damp houses suffering from fungal growth [35,36]. It is a precursor to the formation of 1,3-octadiene. Korpi et al. [18] indicated that microbial air contamination of an indoor environment can be identified when 1-octen-3-ol reaches or exceeds the threshold value, which is equal to 10 µg/m^3^. Some alcohols such as 3-methyl-1-butanol, 4-methyl-1-pentanol and 1-octen-3-ol, can be emitted when fungi grow on media containing carbohydrates. Higher carbon alcohols (1-decanol or 5-hexadecanol) are produced by fatty acid degradation and reduction of amino acids. After only a month of incubation, 4-methyl-1-pentanol and 1-hexanol were identified.

Ketones are produced by fatty acid degradation of, e.g., linoleic acid [37]. Lower carbon ketone (e.g., acetone) is a product of fundamental biochemical processes such, as the Krebs cycle and glycolysis [38]. In turn, unsaturated fatty acids may be transformed into aldehydes, such as octanal, nonanal and decanal, which were found in the largest quantities after two weeks of incubation. Glutaraldehyde was only observed as a species-specific compound for *Poria placenta*.

Phytochemicals (like oxime-methoxy-phenyl or epoxy-linalooloxide) are chemical compounds formed during the metabolic process. These chemicals are often referred to as secondary metabolites. They are generally biologically active in the fungi hosts and play a role in their growth or defense against competitors or pathogens. Carboxylic acids are both substrates and products in the metabolism of MVOCs. Nonanoic acid, n-decanoic acid and tetradecanoic acid are the most abundant acids identified, especially for *Poria placenta* growth. Dimethyl disulphide is produced from the degradation of the sulphur-containing amino acids derived from the malt-extract agar medium [38]. Many other metabolites and metabolic pathways exist, but a detailed description of these is not the main objective of this thesis.

**Figure 4:**
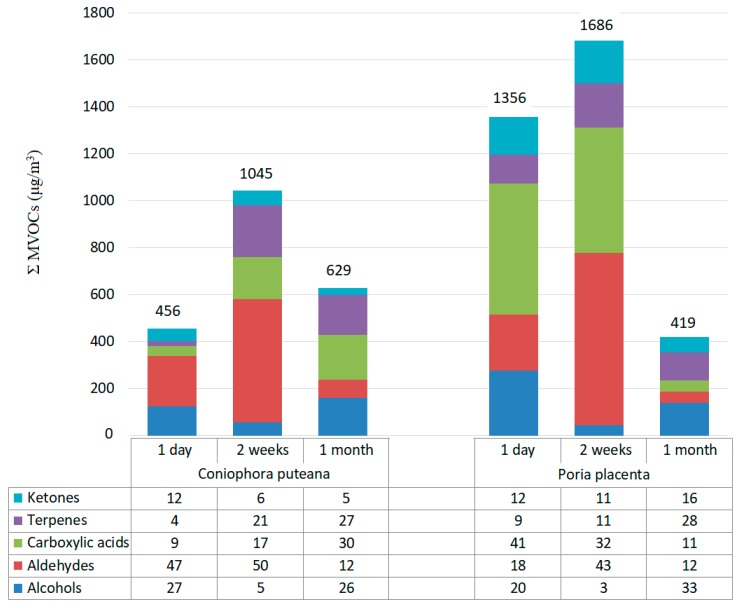
Concentration of Σ MVOCs (μg/m^3^) emitted from *Coniophora puteana* and *Poria placenta* growth on maltose agar medium (numerical values above the bars) and percentage of MVOC constituents for these species throughout the monthly incubation period (table below the chart).

The highest amount of ΣMVOC was observed two weeks after incubation for both fungi species: 1045 μg/m^3^ in the case *Coniophora puteana* and 1686 μg/m^3^ for *Poria placenta*. The sum total of MVOCs emitted from the Kolle flask in which *Poria placenta* was located was equal to 3461 μg/m^3^ (Figure 4). In turn, the sum total of MVOCs emitted from *Coniophora puteana* was equal to 2139 μg/m^3^.

Analysis of the data shows that of all the constituents, aldehydes (up to 50%) and carboxylic acid (up to 41%) were the predominantly identified groups of MVOCs (Figure 4). Nonanal and decanoic acids are compounds present at higher concentrations. Moreover, 0.005% of dimethyl disulphide was identified for *Poria placenta* (not marked on the respective diagram).

### 4.2. Species-Specific MVOCs

Production of MVOCs mainly depends on the growth medium, although many compounds are produced by different species (species-specific compounds) and types of fungi. Significant differences were observed between the compounds produced during growth on paper, building materials and microbiological media [23,24,25,26]. They can develop on organic materials (wood, wallpaper and cardboard), as well as polymeric (expanded polystyrene, mineral wool, plastic film and artificial leather) and mixed materials (carpeting and WPC).

As a result of the described research, 2-ethyl-4-methyl-1-pentanol, 5-hexadecanol, 3-isopropyl-2-phenyl-pent-4-en-2-ol, p-cymene, 2-ethylbutanal, undecanal, 2-ethylhexanoic acid, octadecanoic acid and 1-methyl-2-pyrrolidinone were found to be *Coniophora puteana* species-specific MVOCs. In turn, glutaraldehyde, 1-decanol, 3-hydroxydodecanoic acid, oxime-methoxy-phenyl, 5-methyl-3-hexanol, 3-isopropyl-2-phenyl-pent-4-en-2-ol, 6-methyl-5-hepten-2-one, benzophenone and dimethyl disulphide were found to be *Poria placenta* species-specific MVOCs.

### 4.3. MVOCs Associated with SBS

Damp in residential buildings is a risk factor for the onset of general symptoms and mucosal symptoms. Associations between MVOC and health concerns have been investigated in studies of several countries in Europe and Asia [39,40,41,42]. In the prevalent study, any SBS symptom was associated with some individual volatile organic compounds of possible microbial origin (MVOC) e.g., 2-pentanol, 2-hexanon, 2-pentylfuran, 1-octen-3-ol and 3-methylofuran [43]. The compounds associated with SBS are the same as those found to be associated with the presence of *Basiciomecytes* growing on WPC. The main risk factor for SBS seems to be 1-octen-3-ol. Secondary indicators are 1-hexanol, 2-octen-1-ol and/or dimethyl disulphide, but this finding needs to be collaborated by further investigations.

## 5. Conclusions

The mass spectra of fungal metabolites were obtained by subtracting background (control chamber) spectra from the spectra under investigation (experimental chamber). The authors have demonstrated that the experimental set-up and data analysis method (using subtracted spectra) are capable of measuring MVOCs.

The study of metabolite production resulting from cultures growing on different building materials is of great importance in order to provide information on which metabolites may be expected from WPCs affected by microbial growth. Despite the results obtained, we cannot definitively state the extent to which wood-decay fungi colonize WPC because not all the necessary data were available (e.g., the mass loss rate of the samples and strength parameters). The emissions of MVOCs change with the growth phase.

The largest amount and concentrations of secondary metabolites were identified by both fungi species after two weeks of incubation. The concentration of MVOCs in building materials usually falls within the range 1–50 µg/m^3^. For *Poria placenta*, the most predominant metabolic volatile group identified was carboxylic acids, while aldehydes were the dominant group for *Coniophora puteana*. Terpenes, alcohols and ketones shared similar proportions, and α-pinene, 1-octen-3-ol and acetophenone are the compounds found at higher levels, respectively. Moreover, many compounds were found to overlap for both species, suggesting dependence on the malt-extract agar substrate.

The main indicator of fungal exposure in indoor environments seems to be 1-octen-3-ol, which has also been used as a marker for active growth or damp building materials. Some MVOCs, such as 1-hexanol, 2-octen-1-ol and/or dimethyl disulphide, seem to be positively associated with any SBS symptoms, but epidemiological studies on exposure to MVOC are needed.

## Figures and Tables

**Table 1:** Overview showing the range of MVOC levels emitted by *Coniophora puteana* and *Poria placenta* after one day, two weeks and one month of incubation.

		*Coniophora puteana*			*Poria placenta*		
	Incubation Time	1 Day	2 Weeks	1 Month	1 Day	2 Weeks	1 Month
Volatiles Identified							
Alcohols							
4-Methyl-1-pentanol				+			+
1-Hexanol				++			++
2-Ethyl-4-methyl-1-pentanol				+			
3-Methyl-1-butanol		+	+		+	+	
1-Octen-3-ol		++			++		
2-Octen-1-ol		+	+			+	+
5-Methyl-3-hexanol							+
3-Isopropyl-2-phenyl-pent-4-en-2-ol					+		+
1-Decanol					++		
2-Phenoxy-ethanol				+	++		
5-Hexadecanol				+			
Aldehydes							
Hexanal		+	+	+	+	+	
2-Ethyl-butanal			+				
Heptanal		+	+		+	++	+
Octanal		+	++	+	+	++	
Glutaraldehyde					+	+	+
Nonanal		+	+++	+	+	+++	
Decanal		++	+++			+++	
Undecanal		+	+				
Dodecanal			+			+	
13-Methyltetradecanal						+	
Carboxylic acids							
Pentanoic acid			+		+	+	
Hexanoic acid			+			+	
3-Hydroxydodecanoic acid				+			
2-Ethylhexanoic acid		+					
Nonanoic acid		+	+	+	++	++	
3-Hydroxydodecanoic acid						+	
n-Decanoic acid		+	+	++	+++	+++	
Dodecanoic acid				++		++	
Tridecanoic acid			+			+	
Tetradecanoic acid				+	+++	+++	+
Octadecanoic acid			++				
Terpenes							
alpha-Pinene		+	+++	+++	+	++	++
Vanillin			+			+	
3-Carene		+	++	+	+	+	+
p-Cymene				+			
M-Pyrol					++		
Epoxy-linalooloxide			+			+	
Oxime-methoxy-phenyl						+	
Ketones							
Acetone		+	+	+	+	+	+
3-Methyl-2-cyclopenten-1-one				+		+	
6-Methyl-5-hepten-2-one					+	+	
1-Methyl-2-pyrrolidinone				+			
6,10-Dimethyl-5,9-undecadien-2-one			++			++	
Benzophenone							+
Acetophenone		+			+++	++	+
Sulphur compound							
Dimethyl disulphide						+	

Indicative concentrations of MVOCs (µg/m^3^) are shown as (+) for concentrations below 50 µg/m^3^ (++) for concentrations between 50–100 µg/m^3^ and (+++) for those above 100 µg/m^3^.

**Table 2:** List of MVOCs with types of odors for some identified compounds [8,22].

CAS No.	Compound Name	Odor
66-25-1	Hexanal	fat, tallow, grass
124-13-0	Octanal	green, fat, soap, lemon
112-31-2	Decanal	soap, tallow, orange peel
112-54-9	Dodecanal	fat, citrus, lily
109-52-4	Pentanoic acid	sweet
142-62-1	Hexanoic acid	sweat, fatty, cheesy
3391-86-4	1-Octen-3-ol	earthy, “mushroomy”
22104-78-5	2-Octen-1-ol	green lemon, melon
123-51-3	3-Methyl-1-butanol	truffle
112-05-0	Nonanoic acid	green, fat
334-48-5	Decanoid acid	fat, rancid
143-07-7	Dodecanoid acid	metal
80-56-8	Alpha-Pinene	solvent
121-33-5	Vanillin	vanilla
119-61-9	Benzophenone	almond, burnt sugar
98-86-2	Acetophenone	flower, musty, almond
624-92-0	Dimethyl disulphide	cabbage, onion, putrid
